# Nutritive and Phytochemical Composition of Aromatic Microgreen Herbs and Spices Belonging to the Apiaceae Family

**DOI:** 10.3390/plants11223057

**Published:** 2022-11-11

**Authors:** Maria Giordano, Spyridon A. Petropoulos, Marios C. Kyriacou, Giulia Graziani, Armando Zarrelli, Youssef Rouphael, Christophe El-Nakhel

**Affiliations:** 1 Department of Agricultural Sciences, University of Naples Federico II, 80055 Portici, Italy; 2 Department of Agriculture, Crop Production and Rural Environment, University of Thessaly, Fytokou Street, 38446 Volos, Greece; 3 Department of Vegetable Crops, Agricultural Research Institute, Nicosia 1516, Cyprus; 4 Department of Pharmacy, University of Naples Federico II, 80131 Naples, Italy; 5 Department of Chemical Sciences, University of Naples Federico II, 800126 Naples, Italy

**Keywords:** controlled environment, functional food, young leafy greens, ICP-OES, UHPLC-Q-Orbitrap HRMS, phenolic compounds, quinic acid, antioxidant activity

## Abstract

Microgreens represent a new generation of food products, commonly used to garnish and embellish culinary dishes, and recently associated with an increasing interest in their nutraceutical and phytochemical profiles. Four Apiaceae species: *Pimpinella anisum* L. (anise), *Anthriscus cerefolium* L. (chervil), *Carum carvi* L. (caraway), and *Anethum graveolens* L. (dill) were assessed for fresh yield, macro- and microminerals, total chlorophylls, total ascorbic acid, carotenoids, polyphenols, and their antioxidant activity. Anise was the species yielding the most (2.53 kg m^−2^) and having the highest lutein content (18.4 µg g^−1^ dry weight (DW)). Chervil and dill were characterized by the highest total ascorbic acid content (~151 mg AA g^−1^ fresh weight (FW)). The phenolic profile highlighted the presence of five flavonoid derivatives and 12 phenolic acid derivatives, with quinic acid derivatives being the most abundant phenols in the species tested. In addition, anise, caraway, and dill proved to be considerably rich in total polyphenols (~11056 μg g^−1^ DW). Caraway and dill were characterized by the highest antioxidant activity measured by the DPPH and ABTS methods, whereas the FRAP method revealed caraway as having the highest antioxidant activity. Such results highlight the potential of Apiaceae species as an alternative to other families which are commonly used for microgreens production.

## 1. Introduction

The main objective of modern agriculture is to produce food of superior quality, aiming to comply with the widespread scientific awareness and the current needs of consumers. The term quality refers to both the extrinsic and intrinsic characteristics of crop products [1] and is tightly associated with the molecules of the secondary metabolism. Microgreens, as micro-scale innovative specialty crops, are mainly produced from vegetables seeds, but also from cereals, herbs, and wild species; with a short growing cycle ranging from 7 to 21 days [1,2]. As stated by Caracciolo and coworkers [3], these species used for microgreens production belong to diverse botanical families such as Apiaceae (Umbelliferae)*,* Asteraceae (Compositae)*,* Brassicaeae (Cruciferae), etc. Initially, microgreens were used in cooking as colorful greens appreciated for the lively embellishment of culinary dishes. Currently, they are additionally used for improving the nutritional value of the human diet, since they are naturally rich in macro- and micronutrients, carotenoids, vitamin C, E, and K1, as well as phenolic compounds [4]. Some studies have even highlighted their nutritional potential compared to their mature counterparts. For example, El-Nakhel et al. [5], by comparing two lettuce cultivars, harvested at mature and microgreen stages, it was shown that the microgreen samples possessed a better nutraceutical profile than the mature lettuce samples, in terms of calcium, magnesium, and total polyphenol content. Similarly, Kyriacou et al. [1] reported a higher content of antioxidant molecules and macro- and microminerals in vegetable microgreens compared to their mature counterparts. As for growth, microgreens have a lower photon flux requirement than mature vegetable plants. Therefore, the possibility of growing them in a controlled environment, where the flow of photons and the wavelength can be modulated, makes it possible to regulate the accumulation of phytonutrients and/or reduce the content of antinutrients [6].

In particular, the Apiaceae botanical family includes 434 genera and 3780 species, that are mostly distributed in the northern temperate regions and the high altitudes of tropical regions [7]. The plants belonging to this botanical family have the common characteristic of being aromatic and rich in oils and resins, which are distributed in leaves, roots, stems, and fruits and give particular flavors; allowing these plants to be used in food, drinks, and cosmetics. Species of this family are also rich in secondary metabolites (i.e., coumarins, flavonoids, saponins, steroids, and terpenoids [7]. For example, anise (*Pimpinella anisum* L.) is commonly used to season dishes, due to the aromatic properties of its leaves and seeds [8]. The seeds of this species are also known to have multiple positive effects on human health, having anti-inflammatory, antioxidant, and antimicrobial properties, in addition to their activity against depression and anxiety [9]. Chervil (*Anthriscus cerefolium* L.) Hoffm has been used from ancient times, and is highly appreciated for its diuretic, digestive, and refreshing effect. It is also very useful to relieve stomach and sleep disorders [10]. Caraway (*Carum carvi* L.) is an aromatic plant [11], well known in folk medicine where it is prescribed against digestive disorders, as well as to increase breast milk during breastfeeding [12]. Caraway seeds and fruits have therapeutic properties against inflammation, convulsions, microbial infections, anxiety, hyperglycemia, hormonal alterations of the thyroid, and pneumonia [13]. Finally, dill (*Anethum graveolens* L.) is an annual aromatic plant, widespread in the eastern Mediterranean. It has many therapeutic effects against various diseases of the gastrointestinal tract, against urinary tract infections, as well as in relieving insomnia and rheumatism [14]. It is also used in the food industry as a food flavoring agent [14]. The significant presence of phenolic acids is associated with dill antioxidant and anticancer properties [14].

So far, most qualitative analyses on microgreens have focused on species of the *Brassicaceae* botanical family, as they are rich in polyphenols [15]. However, the growing popularity of microgreens urges for a qualitative characterization of additional species from other families, in order to define their nutritional value and evaluate their potential to improve the human diet. To the author’s best knowledge, no research data are available in the literature covering microgreens cultivation of anise, caraway, and chervil, and the literature on dill is scarce. Caraway, anise, and dill for example were examined for their phenolic profile and antioxidant activity but only as sprouts [16,17,18]. Therefore, based on the aforementioned considerations, these species were assessed in this study as microgreens and characterized for their content of macrominerals (P, K, Ca, Mg, Na), microminerals (Fe, Mn, B, Zn, Se), pigments (total chlorophylls, lutein, *β*-carotene), total ascorbic acid, phenolic profile, and antioxidant activity. 

## 2. Results and Discussion

### 2.1. Growth and Yield

**Figure 1:**
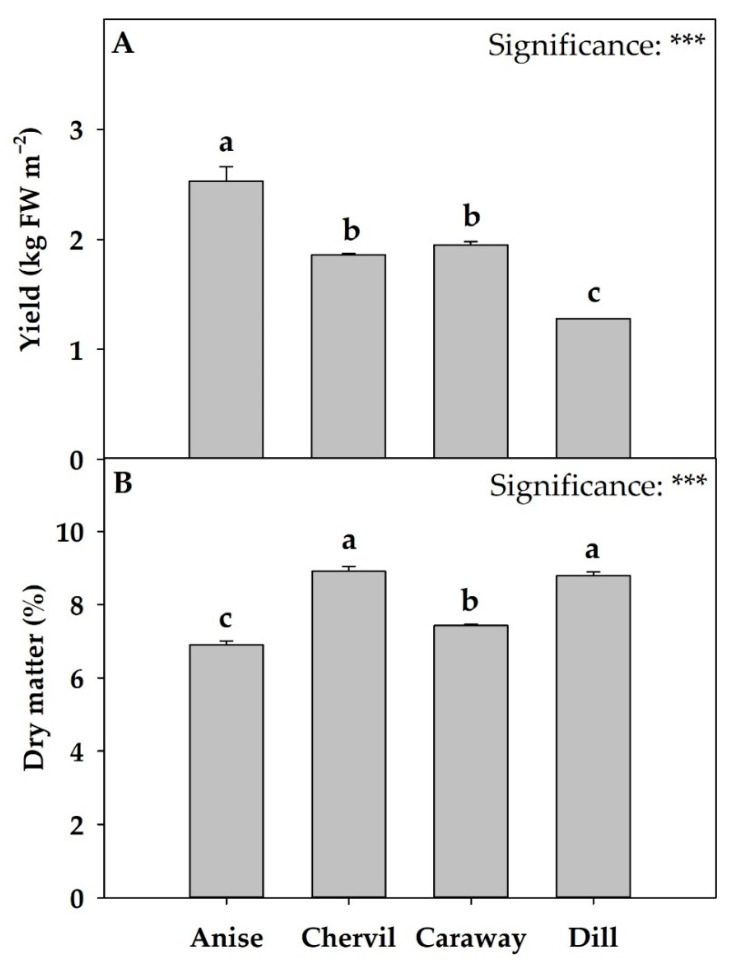
Yield (**A**) and dry matter percentage (**B**) of four Apiaceae microgreens grown under controlled environment. *** indicates the significance at *p* ≤ 0.001. Significant differences based on Tukey’s HSD test are indicated by different letters placed above each vertical bar (*p* = 0.05). All data are expressed as mean ± standard error, n = 3. *Pimpinella anisum* L.: Anise; *Anthriscus cerefolium*: Chervil; *Carum carvi*: Caraway; and *Anethum graveolens*: Dill. FW: fresh weight.

The harvesting stage of microgreens may vary from the cotyledonary stage to the appearance of the first or second true leaf. The harvesting stage, as well as the sowing density, can affect the yield of microgreens [2,4,15]. In our work, the time of appearance and expansion of the first true leaf did not differ among the three species studied (anise, chervil, and dill), which were harvested at 20 days after sowing (DAS), while caraway was harvested at 23 DAS. The recorded fresh yield values ranged between 1.28 and 2.53 kg m^−2^, with significant differences among the species (Figure 1A). The highest yield was recorded for anise, dill produced 49.4% less, while chervil (1.86 kg FW m^−2^) and caraway (1.95 kg FW m^−2^) produced on average 24.7% less than anise. In addition, as presented in Figure 1B the dry matter (DM) values ranged between 6.9 and 8.91%, where the chervil and dill DM% was the highest without significant differences between the two species. 

Kyriacou et al. [4], who examined thirteen species of microgreens belonging to the families of Chenopodiaceae, Malvaceae, Apiaceae, Lamiaceae, and Brassicaceae grown under controlled environment, obtained a range of fresh yield between 1.25 and 5.97 kg m^−2^. Coriander was the only Apiaceae species examined in this report [4] and showed a higher fresh yield (3.30 kg m^−2^) than the species present in our study, while parsley microgreens in the work of El-Nakhel et al. [19] recorded fresh yields within the range of our study (1.37 kg FW m^−2^). These contradictory findings could be attributed to differences in sowing density (e.g., 4–7 seeds cm^−2^ and 6 seeds cm^−2^ in the studies of Kyriacou et al. [4] and El-Nakhel et al. [19], respectively. Such findings also indicate the significant effect of the genetic factor on yield parameters of microgreens. Similar results were also presented in the work of Ghoora et al. [20]. In this particular study, the fresh production of ten microgreens species was examined, including vegetables, leguminous, oleagineous, and aromatic species and the authors reported a wide range of fresh yield values, ranging from 1.12 to 4.93 kg FW m^−2^ [20]. Furthermore, in the same study, it was reported for the two species of Apiaceae examined (*Daucus carota* L. and *Foeniculum vulgare* Mill.), fresh yield values of 2.20 and 1.20 kg m^−2^, respectively, highlighting production differences between species of the same family [20]. Moreover, Kyriacou et al. [4] reported values of dry matter lower than those obtained in our work for the thirteen species of microgreens examined (values between 3.89% and 6.05%), while coriander which belongs to the Apiaceae family recorded one of the highest DM%.

Differences in microgreens production may also be due to the growth medium. For example, Kyriacou et al. [21], following the production of *Coriandrum sativum* L. microgreens, reported different yield values depending on the type of substrate used. In particular, the fresh weight of coriander microgreens was twice as high as when the microgreens were grown on peat-based substrate compared to natural fiber and synthetic substrates, but with lower DM%. Moreover, dill microgreens grown in a floating system in greenhouse conditions [19] were characterized by a lower yield around 0.81 kg FW m^−2^ and higher DM%, which could be explained by the different growing conditions.

### 2.2. Macro- and Microminerals

Microgreens are considered rich sources of minerals [22], that are capable of regulating cell homeostasis and metabolism in the human body, and serve as co-factors for several enzymes [4,16]. In our study, among the macrominerals examined, K was the most abundant in the four Apiaceae microgreen species, while Ca was the second most abundant macromineral (Table 1). Among the species studied, P and K were the least present in dill (7.7 and 21.7 mg g^−1^ DW, respectively). Anise and chervil had a similar Mg content (mean value of 2.26 mg g^−1^ DW), which was significantly higher than caraway and dill (mean value of 1.84 mg g^−1^ DW). Finally, Na was the highest in dill (2.91 mg g^−1^ DW). 

The four microgreen species in this work had on average a higher P and Ca content and a lower Mg and Na content than coriander examined by Kyriacou et al. [4]. Calcium is among the most important minerals, and it is also abundant in several species of microgreens as examined in other papers [4,16]. Parsley microgreens for example grown in similar conditions [19] were characterized by a higher amount of K, Mg, and Na when expressed on a dry weight basis, almost double that found in this study. The content of the macrominerals P, K, and Ca found in our work, was higher than those obtained in two species of Apiaceae microgreens (*Daucus carota* L. and *Anethum graveolens* L.), examined by El-Nakhel et al. [23]. Regarding the above-mentioned macronutrients, our values were also higher than those found in the work of Pannico et al. [24], who examined the mineral profile of four microgreen species (coriander, green basil, purple basil, tatsoi) grown on capillary mat. These authors reported for coriander microgreens the following mineral values: P: 3.04 mg g^−1^ DW; K: 7.23 mg g^−1^ DW; Ca: 3.15 mg g^−1^ DW; Mg: 2.17 mg g^−1^ DW; Na: 0.61 mg g^−1^ DW. While the differences in the mineral profile between varieties of the same species highlight the effect of genetic factors, the differences between different species could be attributed not only to genetic factors, but also to growing conditions, the type of growth substrates used, and the composition of the nutrient solution [6]. Moreover, Kyriacou et al. [21] demonstrated that substrate choice greatly influence the macromineral accumulation of coriander. Moreover, the minerals found in microgreens could be regulated based on certain consumer needs through the management of the nutrient solution [2,22]. For example, a low sodium content is important to counteract high blood pressure and strokes [25]. Finally, harvesting stage is also important since different growth stages exhibit different root growth and consequently different mineral uptake and accumulation in plant tissues [26].

Regarding the microminerals of anise, chervil, caraway, and dill (Table 2), Fe, Mn and Z were the most abundant minerals. Mn proved to be particularly high in chervil, almost 94.1% higher than the other three species. Moreover, no significant differences were found among the four species in the case of Se, which had an average value of 1.51 µg g^−1^ DW. Significant differences were found for B (the fourth most abundant micromineral), which had the lowest value in caraway (21.7 µg g^−1^ DW). Coriander microgreens examined by Pannico et al. [24], showed values of 20.45 µg g^−1^ DW, 23.18 µg g^−1^ DW, 6.61 µg g^−1^ DW for Fe, Zn, Mn, respectively, which were lower than those recorded in our work. As for dill microgreens in an experiment run by Kusumitha et al. [27], Fe and Ca registered 3.98 and 52.12 mg 100 g^−1^ FW, respectively. Se concentration in anise, chervil, caraway, and dill was much higher, almost 10-fold than that registered in carrot and fennel microgreens mentioned by Ghoora et al. [20]. Carrot microgreens in the same work showed similar concentration of Fe but more zinc compared to the Apiaceae species studied in this work, while fennel microgreens showed an opposite trend, with similar Zn concentration and more Fe. Microminerals are fundamental for cellular metabolism, both in plants and in humans. They are involved in many metabolic pathways involving proteins, enzymes, and hormones, thus being essential for many biological functions [28]. They also contribute to the antioxidant activity of plants because they are involved in the metabolism of antioxidant molecules and enzymes [29]. Deficiencies in some micronutrients, such as Se, Zn, and Fe, result in a form of malnutrition widespread in the world, known as hidden hunger, which causes human infection, heart disease, inflammation, and various types of cancer [30]. Modern science is engaged in fighting this malnutrition through different biofortification strategies of crops with microminerals, while microgreens are ideal for this purpose [29].

### 2.3. Chlorophylls, Carotenoids, and Total Ascorbic Acid

Microgreens are defined as super-foods or functional foods for their content of vitamins E, C, and K, carotenoids (β-carotene, lutein, and zeaxanthin), and their antioxidant activity which is almost ten times greater than that of mature leafy vegetables [2]. The advantage of microgreens is that they are eaten raw, so they maintain thermolabile vitamins, such as vitamin C [2]. The four varieties examined in our study did not significantly differ in the content of total chlorophylls, having an average value of 1.037 mg g^−1^ FW (Table 3). Anise was among the species with the highest content of lutein (18.4 µg g^−1^ DW), whereas the lowest β-carotene concentration was recorded in dill microgreens. Finally, dill and chervil had similar total ascorbic acid content (~151.0 mg 100 g^−1^ FW), which was higher than that found in anise and caraway (Table 3).

El-Nakhel et al. [23] reported total carotenoid content values equal to 0.316 mg g^−1^ FW for *Daucus carota* L. and *Anethum graveolens* L. microgreens. The same authors also recorded similar values for total chlorophylls in dill. In addition, the values obtained of lutein and β-carotene are in line with those obtained in a previous work on parsley grown in the same conditions [19]. Kusumitha and coworkers [27] found that dill microgreens exhibited a 3.35 mg 100 g^−1^ FW of β-carotene. Moreover, Kyriacou et al., [4] detected a variable lutein content from 193.5 mg kg^−1^ DW (mustard microgreens) to 827.9 mg kg^−1^ DW (in jute microgreens), with coriander microgreens recording a lutein content of 391.9 mg kg^−1^ DW. Moreover, lutein and β-carotene detected in coriander in a different study, were as well in a different range which indicates that carotenoid content depends not only on the species of microgreens but also on the growing conditions [24,31]. In addition, the results of β-carotene in this experiment were lower almost 5-fold and 9-fold than that found in carrot and fennel microgreens [20]. Carotenoids present in leafy vegetables are known to reduce the risk of macular degeneration and cataracts [19,23], while its dietary intake is considered more beneficial to human health compared to an intake through supplements [5]. Equally, chlorophylls are deemed beneficial to human wellbeing, by chelating heavy minerals and collaborating in the neutralization of free radicals [5]. Regarding total ascorbic acid, the literature reports for other Apiaceae species values of 130.9 mg 100 g^−1^ FW for coriander [4], 20.26 mg 100 g^−1^ FW for parsley [19], 70.35 mg 100 g^−1^ FW for carrot and 137.8 mg 100 g^−1^ FW for dill [23], 46 mg 100 g^−1^ FW [28], and 52.5 mg 100 g^−1^ FW for fennel [20]. The values measured for the four species in the present study are higher than those recorded for lettuce microgreens [5] but significantly lower than those registered for other species [4]. Considering the daily vitamin C intake levels (60 mg) recommended for an adult by the European Food Safety Authority (EFSA) [2], the microgreens from our work can satisfy this value with the consumption of 40 g of chervil/dill, 64 g of anise, or 83 g of caraway.

### 2.4. Phenolic Profile

Microgreens are considered functional foods due to their high polyphenol content, which contributes by its diverse categories to the secondary metabolism and functioning of the plants [6]. In microgreens, polyphenols can represent a quality attribute that can guide consumer choice and increase the added value of the final product [2,32].

Seventeen phenolic compounds were detected by the Q Exactive Orbitrap LC–MS/MS analysis in the four Apiaceae microgreens examined. These compounds were represented by 12 phenolic acids and five flavonoids, including flavonols and flavones (Table 4). Anise had the highest total polyphenol content with values of 11,450 µg g^−1^ DW, followed by dill (11249 µg g^−1^ DW), caraway (10,471 µg g^−1^ DW), and finally chervil with the lowest content of total phenols (1970 µg g^−1^ DW). Moreover, the Apiaceae microgreens tested, differed significantly in the content of individual phenolic compounds. However, in all the studied species, caffeoyl quinic acid was the most abundant compound with values of 7995, 7040, 5447, 948 µg g^−1^ DW for dill, anise, caraway, and chervil, respectively (Table 4). The same decreasing order was recorded for feruloyl quinic acid with values of 2977, 2937, 1761, 491 µg g^−1^ DW, for dill, anise, caraway, and chervil, respectively. Another abundant phenolic compound was dicaffeoyl quinic acid with the highest value of 3168 µg g^−1^ DW recorded in caraway, being 6.7-fold higher than the other three species. The detected flavonoids included five compounds with quercetin rhamnoside being the most abundant one in anise. Regarding the differences observed between the tested species, anise differed from the other species by being rich in salicylic acid hexoside (150.4 µg g^−1^ DW), dihydroferulic acid (71.4 µg g^−1^ DW), caffeoyl shikimate acid (6.78 µg g^−1^ DW), coumaroyl diglucoside (3.01 µg g^−1^ DW), luteolin-7-O-glucoside (35.9 µg g^−1^ DW), quercetin rhamnoside (125 µg g^−1^ DW), and coumaric acid (7.29 µg g^−1^ DW), when compared to the other three species. In addition, anise was characterized by the presence of vanillic acid and apigenin-7-O-glucoside (data not shown), while dill was the species with the highest content of kaempferol-3-dihexoside (14.5 µg g^−1^), sinapinic acid hexose (21.6 µg g^−1^ DW), hyperoside (56.4 µg g^−1^ DW), and rutin (49.8 µg g^−1^ DW). Finally, chervil differed from the other microgreens tested by showing the highest content of ferulic acid (68.7 µg g^−1^ DW) (Table 4).

Polyphenols belonging to the class of flavonoids, such as anthocyanins, flavanols, and flavones, together with phenolic acids are important secondary metabolites for plant protection against biotic and abiotic stressors, while they exert significant antioxidant properties which are important for human health [33]. The quantitative and qualitative analyses of the phenolic profile of the species examined in our work, revealed a higher total phenolic content than that found for the microgreens of Lamiaceae, Brassicaceae, Amaranthaceae, Chenopodiaceae, and Malvaceae botanical families, reported in the literature [4,15]. Moreover, the phenolic profile of coriander microgreens studied by Xiao et al. [32] included quercetin 3-O-rutinosides (rutin), kaempferol, and luteolin as the main phenolic compounds, with values of 2392.2 μg g^−1^ DW, 1315.7 μg g^−1^ DW, and 1469.6 μg g^−1^ DW, respectively. Quercetin-3-O-rutinoside and quercetin-3-O-glucuronide were also the most abundant phenolic compounds for mature coriander plants in the work of Barros et al. [34], with values of 3296.2 μg g^−1^ DW and 1237.1 μg g^−1^ DW, respectively. These results highlight the high variability of the content of phenolic compounds as well as in the profile of individual phenolic compounds among different species or varieties of the same species, while the harvesting stage and growing conditions are also important factors that may affect phenolic compound composition [14,21]. In a different stage of harvest such as sprouts in the work of Balenescu et al. [18], anise was demonstrated to have higher total polyphenol and total flavonoid content than dill sprouts, similar to our harvest stage. Harakotr et al. [35] found that dill microgreens were characterized by a total phenolic content of ~10 mg GAE g^−1^ DW and a total flavonoid content of ~1000 mg CE g^−1^ DW. Jana and Shekhawat [36], examined mature dill plants and found significant differences in the content of tannins, terpenoids, and flavonoids in relation to plant parts (leaves, roots, stems, seeds) and the solvent of extraction (water or ethanol). Furthermore, the concentration and antimicrobial efficacy of these compounds were related to the agricultural practices adopted during cultivation.

### 2.5. Antioxidant Activity

**Figure 2:**
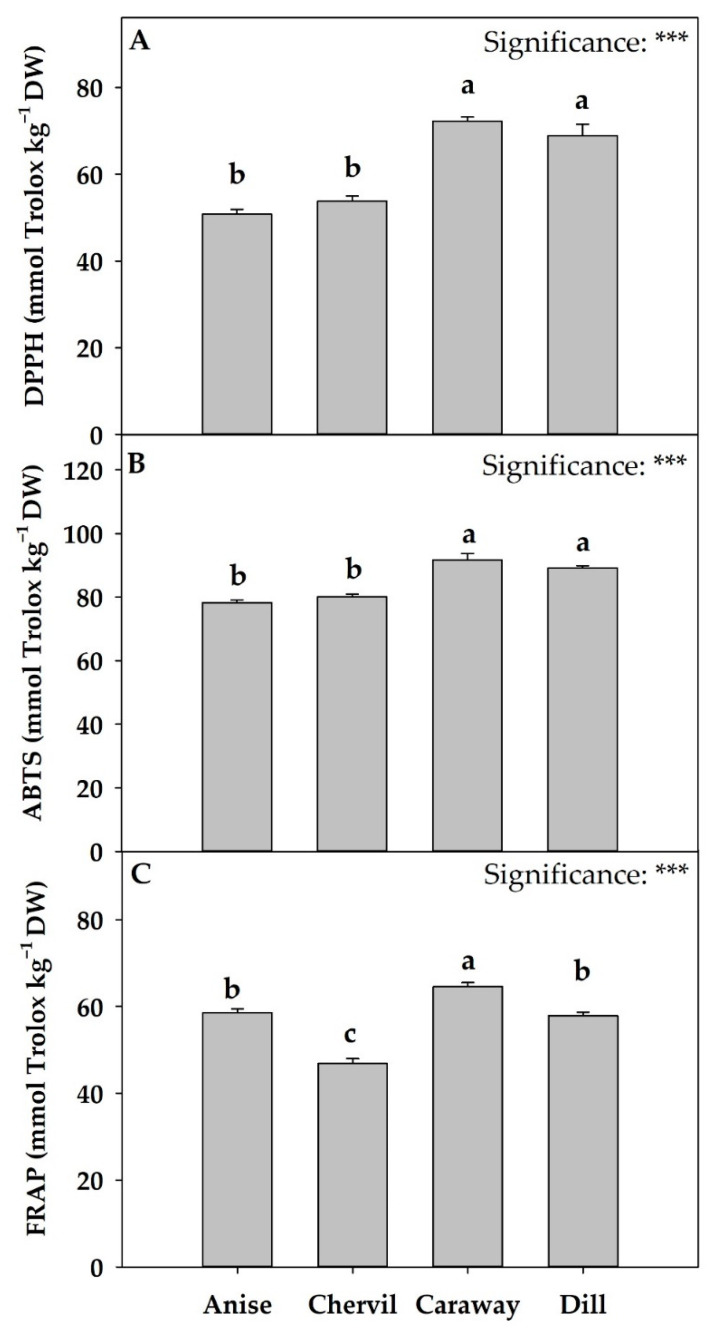
Antioxidant activity DPPH (**A**), ABTS (**B**), and FRAP (**C**) of four Apiaceae microgreens grown in controlled environmental conditions. *** indicates significance at *p* ≤ 0.001. Significant differences based on Tukey’s HSD test are indicated by different letters placed above each vertical bar (*p* = 0.05). All data are expressed as mean ± standard error, n = 3. *Pimpinella anisum* L.: Anise; *Anthriscus cerefolium*: Chervil; *Carum carvi*: Caraway; and *Anethum graveolens*: Dill. DW: dry weight.

The results of the antioxidant activity of the four species were determined with three different assays and are presented in Figure 2. Based on the DPPH (Figure 2A) and ABTS method (Figure 2B), caraway and dill exhibited the highest antioxidant activity compared to anise and chervil. Caraway was also the species with the highest antioxidant activity, based on the FRAP method (Figure 2C), being significantly different from anise, dill, and chervil. Similarly, dill microgreens in a different research study [33], demonstrated similar ABTS and DPPH antioxidant activity in light intensity conditions close to ours. According to the literature, the antioxidant activity (expressed in mmol Trolox kg^−1^) in thirteen species of microgreens examined by Kyriacou et al. [4], varied between 303.3 mmol Trolox kg^−1^ DW in jute and 878.3 mmol Trolox kg^−1^ DW in cress, while values of 616.2 mmol Trolox kg^−1^ DW were recorded for the only Apiaceae species examined, namely coriander. The variable results of antioxidant activity reported in the literature with those of our study could be attributed to different growing conditions which may induce the biosynthesis of secondary metabolites, since the antioxidant activity of aromatic species, such as those belonging to the Apiaceae family, is usually associated with total phenol content [14] as well as to ascorbic acid content, which is a powerful antioxidant both in plants and in the human body [5]. In any case, the variability of antioxidant activity among different cultivars or species of Apiaceae plants was related to different genotypes, methods of extraction, growth conditions, and fertilization [14]. 

## 3. Materials and Methods

### 3.1. Plant Material and Growth Condition

Seeds of the four species of Apiaceae/Umbelliferae (*Pimpinella anisum* L.: anise; *Anthriscus cerefolium*: chervil; *Carum carvi*: caraway; and *Anethum graveolens*: dill, “Pagano Costantino & F.lli SRL Scafati, Salerno, Italy) were sown in plastic trays (204 cm^2^) at a density of 6 seeds cm^−2^, except for anise where a density of 7 seeds cm^−2^ was adopted. Each plastic tray was filled with a peat-based substrate (650 cm^3^). The substrate physicochemical properties and the growing conditions were previously described in the work of Petropoulos et al. [37]. Plants were grown in a controlled environment, where temperature, humidity, light, and nutrient supply were kept constant throughout the growing cycle. A daily fertigation with a quarter-strength Hoagland’s nutrient solution was applied [38]. A completely randomized design was adopted, with three replicates for each microgreen species.

### 3.2. Sampling of Microgreens, Fresh Weight and Dry Matter Determination

Microgreens of the different species were harvested at the first true-leaf stage, at 20 days after sowing (DAS), except for caraway which was harvested at 23 days DAS. Harvesting was performed using scissors to cut the plants at the substrate level without picking up impurities from the substrate. Immediately after harvest, the samples were weighed for the determination of fresh weight (g) and yield calculation which was expressed as kg m^−2^. A sub-sample was isolated from each fresh sample to be cold-lyophilized in order to determine antioxidant activities, carotenoids (ß-carotene and lutein), and phenolic compounds. The remaining part of each freshly collected sample was frozen in a part at −80 °C and subsequently used for the assessment of total chlorophylls and total ascorbic acid. Another sub-sample was used for dry weight (DW) determination after drying at 65 °C until constant weight. The dry matter (DM) of microgreens was calculated by dividing the dry weight by the yield, and then expressed as a percentage. The dry samples were used for the determination of macro- and microminerals.

### 3.3. Macro- and Micromineral Analysis by ICP-OES

The quantification of macro- and microminerals was performed by inductively coupled plasma-optical emission spectrometry (ICP-OES, Spectroblue, Spectro Ametek, Berwyn, PA, USA) [39]. In brief, dried microgreens were ground in a Wiley Mill (841 µm screen) and 1 g of each dry sample was digested in a microwave digestion system (MLS-1200 Microwave Laboratory Systems, Milestone, Shelton, CT, USA) in 10 mL of nitric acid (65%) and hydrochloric acid (37%) (3:1 *v*/*v*). After digestion, the resulting solutions were transferred to 100 mL volumetric flasks and diluted with ultra-pure water (Milli-Q, Merck Millipore, Darmstadt, Germany) to the fixed volume. An aliquot of this solution was used to quantify the minerals. For Fe, Mn, B, Zn, and Se, the values were presented as µg g^−1^ DW. For macrominerals and Na, the values were presented in mg g^−1^ DW. The calibration curve was prepared using a working standard solution with concentrations ranging from 1.0 to 100 µg L^−1^ for all non-alkaline minerals (P, Fe, Zn, Mn, B, Se) and with concentrations ranging from 100 µg L^−1^ to 10 mg L^−1^ for all alkaline minerals (K, Ca, Mg, Na). 

### 3.4. Analysis of Chlorophylls, Carotenoids, and Total Ascorbic Acid

Total chlorophylls were determined spectrophotometrically with a Hach DR 2000 spectrophotometer (Hach Co., Loveland, CO, USA). An amount of 500 mg of each fresh microgreen sample was extracted with 10 mL 90% ammoniacal acetone (Carlo Erba Reagents S.r.l., Milan, Italy), and left in darkness for 15 min. Then, the extracts were centrifuged at 2000× *g* for 10 min. The supernatant was diluted with 90% ammoniacal acetone to reach a final volume of 25 mL. To determine the content of chl. a and b, the absorbance was read at 663 nm and 647 nm wavelengths with a UV-Vis spectrophotometer (DR2000, Hach Co., Loveland, CO, USA), respectively, and the values were calculated using the formulas and extinction coefficients proposed by Lichtenthaler and Buschmann [40]. 

The determination of lutein and β-carotene was performed by HPLC-DAD according to the method of Kim et al. [41]. In brief, 100 mg of freeze-dried and ground microgreens was diluted with 6 mL of ethanol containing 0.1% BHT. The extraction took place at 85 °C in a water bath for 5 min and then 120 μL of 80% KOH was added to each solution. The samples were vortexed and returned to the water bath for 10 min for saponification. After that, 3 mL of hexane and 3 mL of distilled water were added, and then the samples were immediately put in ice. The centrifugation enabled the formation of a hexane layer containing the crude carotenoids. The residual pellet was extracted again with hexane, and the hexane layers were mixed and dried with nitrogen gas. The residue was dissolved in 1 mL of chloroform and filtered (0.2 µm nylon filter). For the quantification, a reverse phase-HPLC separation was undertaken with a Shimadzu HPLC LC 10 (Shimadzu, Osaka, Japan) equipped with a 250 × 4.6 mm, 5 µm Gemini C18 column (Phenomenex, Torrance, CA, USA) according to conditions previously described by Kyriacou et al. [4]. In brief, 20 µL of each sample was injected and eluted with acetonitrile as mobile phase A and ethanol:n-hexane:dichloromethane (1:1:1) as phase B at a flow rate of 1 mL min^−1^. A gradient of mobile phases A and B was built over a 25 min run as follows: 0–8 min (82:18); 8–12 min (76:24); 12–18 min (39:61); and 18–25 min a linear gradient from 39:61 to 82:18 for equilibration. The absorbance of the eluent was measured at 450 nm. Authentic lutein and β-carotene were used to evaluate their quantity in the sample based on external calibration curves ranging from 5–100 μg mL^−1^ including a minimum of six levels of concentration.

Total ascorbic acid content was determined by UV-Vis spectrophotometer (Hach DR 4000; Hach Co, Loveland, CO, USA), according to the method of Kampfenkel, et al. [42] The frozen microgreen samples (400 mg) were ground in 2 mL of 6% TCA. After centrifugation, a 200 µL aliquot of the supernatant was incubated with dithiothreitol. The addition of ferric chloride allowed the reduction of Fe^3+^ to Fe^2+^, which is then complexed with 2,2-dipyridyl. This complex has an absorption at a wavelength of 525 nm.

### 3.5. Phenolic Profile by UHPLC-Q-Orbitrap HRMS

To determine phenolic profile and total polyphenols (total phenolic acid derivatives and total flavonoid derivates), 100 mg of each lyophilized sample was mixed with 5.0 mL of methanol/water (60:40 *v*/*v*) and sonicated for 30 min at room temperature (Kyriacou et al., 2019). The extraction protocol and the equipment used for the analysis of phenolic compounds was previously described by Kyriacou et al. [4]. The limit of detection (LOD), limit of quantification (LOQ), linearity of calibration curves, recovery percentage (%), and intra- and inter-day precision of the equipment are presented in Supplementary Material Table S1.

The phenolic profile was determined using ultrahigh-performance liquid chromatography-quadrupole-Orbitrap high-resolution mass spectrometry (UHPLC-Q-Orbitrap HRMS; Thermo Fisher Scientific, Waltham, MA, USA) according to the protocol previously described by Petropoulos et al. [37] and the results are presented as µg g^−1^ DW.

### 3.6. Antioxidant Activity

The antioxidant activity was evaluated by UV–Vis spectrophotometry with three different methods by extracting the samples of lyophilized microgreens with methanol and taking an aliquot of the extract for each method. The 2,2-diphenyl-1-picrylhydrazyl (DPPH)-based method involved the preparation of a solution of 4 mg of DPPH in 10 mL of methanol (96%) [43]. Then an aliquot of 200 µL of extract from each sample of microgreens was incubated at room temperature for 10 min with 1 mL of DPPH solution and measured at 517 nm. 

The ferric reducing antioxidant activity (FRAP method; [44]) was determined by adding 150 µL of the extract of each sample of microgreens in 2.850 mL of FRAP working solution at room temperature for 4 min, and subsequent reading of the absorbance at 593 nm. The 2,2-azinobis- (3-ethylbenzothiazoline-6-sulfonate) (ABTS) method involved the reaction of ABTS.+ radicals with the extract of each sample [45]. The absorbance readings were conducted at 734 nm. 

The results for all the assays were presented as Trolox Equivalent Antioxidant Activity, mmol Trolox equivalents kg^−1^ DW.

### 3.7. Statistical Analysis

The analysis of data was performed using the one way-ANOVA by SPSS 20 software package. The differences between species for each parameter were evaluated according to Tukey’s HSD test (*p* = 0.05). All the data are presented as mean ± standard error, n = 3.

## 4. Conclusions

The cultivation of microgreens is becoming increasingly popular, and the demand for new species with high nutraceutical profile and organoleptic traits is growing. This study highlighted the effect of the genotype on the growth and quality of four Apiaceae microgreens, with significant differences being recorded. All the species showed promising results in terms of yield, especially anise which recorded the highest fresh yield. Moreover, the studied microgreens could be considered rich sources of macro and micro-nutrients although significant differences were recorded between them, especially caraway which contained the highest amounts of phosphorus, potassium, and iron. In regard to antioxidant compounds, dill microgreens were the richest in total ascorbic acid, while anise showed the highest carotenoid and lutein content. The phenolic profiling revealed the dominance of quinic acid derivates for all the species studied, and caraway showed the highest antioxidant activity regardless of the assay tested. In conclusion, our results highlighted the potential of Apiaceae species as an alternative to other families which are commonly used for microgreens production. The high variability in chemical composition recorded in our study, as well as the numerous aromatic and medicinal species of the Apiaceae family, highlight the importance of studying more species with the aim to diversify our daily diet with healthy and functional microgreens.

## Figures and Tables

**Table 1:** Macromineral content of four Apiaceae species of microgreens grown in controlled environment conditions.

Species	P	K	Ca	Mg	Na
	mg g^−1^ DW				
Anise	10.9 ± 0.31 a	27.7 ± 0.54 ab	12.6 ± 0.50 b	2.28 ± 0.11 a	0.18 ± 0.02 d
Chervil	9.27 ± 0.38 ab	23.8 ± 0.63 bc	13.0 ± 0.25 b	2.24 ± 0.04 a	0.76 ± 0.02 b
Caraway	10.1 ± 0.64 a	29.9 ± 1.00 a	9.6 ± 0.10 c	1.88 ± 0.05 b	0.31 ± 0.01 c
Dill	7.17 ± 0.23 b	21.7 ± 1.00 c	18.0 ± 0.71 a	1.81 ± 0.08 b	2.91 ± 0.04 a
Significance	***	**	***	**	***

** and *** indicate significance at *p* ≤ 0.01, and 0.001, respectively. Significant differences based on Tukey’s HSD test are indicated by different letters placed within each column (*p* = 0.05). All data are expressed as mean ± standard error, n = 3. *Pimpinella anisum* L.: Anise; *Anthriscus cerefolium*: Chervil; *Carum carvi*: Caraway; and *Anethum graveolens*: Dill. DW: dry weight.

**Table 2:** Micromineral content of four Apiaceae microgreens species grown in controlled environmental conditions.

Species	Fe	Mn	B	Zn	Se
	µg g^−1^ DW				
Anise	57.0 ± 1.8 a	37.6 ± 0.7 c	25.3 ± 0.8 ab	47.5 ± 0.5 a	1.75 ± 0.16
Chervil	45.6 ± 1.8 b	78.5 ± 0.5 a	24.8 ± 0.2 bc	47.0 ± 0.9 a	1.40 ± 0.07
Caraway	59.9 ± 0.7 a	43.8 ± 0.8 b	21.7 ± 0.4 c	32.2 ± 1.4 b	1.44 ± 0.15
Dill	34.5 ± 1.7 c	40.1 ± 0.8 bc	28.3 ± 1.2 a	27.0 ± 0.5 c	1.47 ± 0.14
Significance	***	***	**	***	ns

Non-significant (ns), ** and *** indicate significance at *p* ≤ 0.01 or 0.001, respectively. Significant differences based on Tukey’s HSD test are indicated by different letters placed within each column (*p* = 0.05). All data are expressed as mean ± standard error, n = 3. *Pimpinella anisum* L.: Anise; *Anthriscus cerefolium*: Chervil; *Carum carvi*: Caraway; and *Anethum graveolens*: Dill. DW: dry weight.

**Table 3:** Total chlorophylls, carotenoids, and total ascorbic acid (TAA) concentrations of four Apiaceae microgreens grown in controlled environment conditions.

Species	Total Chlorophylls	Lutein	*β*-Carotene	TAA
	mg g^−1^ FW	µg g^−1^ DW		mg AA 100 g^−1^ FW
Anise	1.10 ± 0.08	18.4 ± 0.8 a	172 ± 1.8 a	94.5 ± 2.1 b
Chervil	1.01 ± 0.04	13.1 ± 0.5 b	170 ± 0.7 a	152 ± 1.4 a
Caraway	1.05 ± 0.03	13.9 ± 1.1 b	179 ± 1.5 a	71.6 ± 0.9 c
Dill	0.99 ± 0.04	8.81 ± 0.2 c	126 ± 2.7 b	150 ± 2.4 a
Significance	ns	***	***	***

Non-significant (ns), *** indicates significance at *p* ≤ 0.001, respectively. Significant differences based on Tukey’s HSD test are indicated by different letters placed within each column (*p* = 0.05). All data are expressed as mean ± standard error, n = 3. *Pimpinella anisum* L.: Anise; *Anthriscus cerefolium*: Chervil; *Carum carvi*: Caraway; and *Anethum graveolens*: Dill. DW: dry weight, FW: fresh weight.

**Table 4:** Phenolic profile of four Apiaceae microgreens grown in controlled environment conditions.

Phenolic Compounds (μg g^−1^ DW)	Species				Significance
	Anise	Chervil	Caraway	Dill	
PHENOLIC ACID DERIVATIVES					
salicylic acid hexoside	150.4 ± 1.0 a	36.8 ± 0.3 c	44.3 ± 0.8 b	16.9 ± 1.5 d	***
caffeic acid hexoside	4.59 ± 0.21 a	4.81 ± 0.05 a	4.21 ± 0.37 a	1.42 ± 0.10 b	***
dihydroferulic acid	71.4 ± 1.2 a	5.75 ± 0.08 b	3.25 ± 0.21 b	4.87 ± 0.28 b	***
sinapinic acid hexose	4.18 ± 0.04 d	12.9 ± 0.59 b	7.54 ± 0.64 c	21.6 ± 0.32 a	***
feruloyl quinic acid	2937 ± 0.5 b	491 ± 0.3 d	1761 ± 0.88 c	2977 ± 2.3 a	***
caffeoyl quinic acid	7040 ± 1.2 b	948 ± 1.2 d	5447 ± 1.1 c	7995 ± 3.5 a	***
dicaffeoyl quinic acid	950.9 ± 0.9 b	396.7 ± 3.6 c	3168.1 ± 2.62 a	61.2 ± 0.92 d	***
caffeic acid	1.119 ± 0.140	1.228 ± 0.050	0.961 ± 0.027	1.234 ± 0.080	ns
caffeoyl shikimate acid	6.78 ± 0.26 a	0.19 ± 0.01 c	0.64 ± 0.06 bc	0.85 ± 0.06 b	***
coumaroyl diglucoside	3.01 ± 0.11 a	0.64 ± 0.09 b	0.22 ± 0.03 c	0.28 ± 0.08 bc	***
ferulic acid	42.6 ± 1.4 b	68.7 ± 0.7 a	17.8 ± 0.7 c	44.4 ± 0.7 b	***
coumaric acid	7.29 ± 0.53 a	1.30 ± 0.17 c	3.77 ± 0.64 b	1.08 ± 0.08 c	***
Total phenolic acid derivatives	11,218.50 ± 1.96 a	1967.82 ± 3.61 d	10,459.28 ± 1.95 c	11,126.23 ± 6.11 b	***
FLAVONOID DERIVATIVES					
rutin	32.7 ± 1.09 b	0.28 ± 0.01 c	1.13 ± 0.05 c	49.8 ± 0.81 a	***
hyperoside	27.5 ± 0.94 b	0.19 ± 0.02 d	7.87 ± 0.44 c	56.4 ± 0.90 a	***
luteolin-7-O-glucoside	35.9 ± 0.53 a	0.25 ± 0.00 b	0.52 ± 0.00 b	0.29 ± 0.00 b	***
quercetin rhamnoside	125 ± 1.8 a	1.61 ± 0.02 b	1.92 ± 0.02 b	1.82 ± 0.08 b	***
kaempferol-3-dihexoside	10.3 ± 0.83 b	0.10 ± 0.04 c	0.31 ± 0.05 c	14.5 ± 0.31 a	***
Total flavonoid derivatives	231.57 ± 4.53 a	2.44 ± 0.07 c	11.77 ± 0.47 c	122.85 ± 1.46 b	***
TOTAL POLYPHENOLS	11,450.07 ± 3.01 a	1970.26 ± 3.65 d	10,471.05 ± 2.13 c	11,249.10 ± 6.07 b	***

Non-significant (ns), *** indicates significance at *p* ≤ 0.001, respectively. Significant differences based on Tukey’s HSD test are indicated by different letters placed within each column (*p* = 0.05). NF = not found. All data are expressed as mean ± standard error, n = 3. *Pimpinella anisum* L.: Anise; *Anthriscus cerefolium*: Chervil; *Carum carvi*: Caraway; and *Anethum graveolens*: Dill. DW: dry weight.

## Data Availability

The datasets generated for this study are available on request to the corresponding author.

## Supplementary Materials

The following supporting information can be downloaded at: https://www.mdpi.com/article/10.3390/plants11223057/s1, Table S1: Limit of detection (LOD), limit of quantification (LOQ), linearity of calibration curves, recovery percentage (%) and intra- and inter-day precision of the Ultra High Pressure Liquid Chromatograph used for phenolic compounds detection.
