# Supplementary material for: Nutritive and Phytochemical Composition of Aromatic Microgreen Herbs and Spices Belonging to the Apiaceae Family
Source: Plants (Basel). 2022 Nov 11;11(22):3057. doi: 10.3390/plants11223057 (PMC9695664; doi:10.3390/plants11223057)
Supplement: Supplementary file 1 [file plants-11-03057-s001.zip › plants-2013214-supplementary.pdf]

Table S1. Limit of detection (LOD), limit of quantification (LOQ), linearity of calibration curves, recovery percentage (%) and intra- and inter-day precision of the Ultra High Pressure Liquid Chromatograph used for phenolic compounds detection.

COMPOUNDS	LOD	LOQ	Linearity	Recovery (%)			Intra-Day Precision (RSD, %; n=3)	Inter-Day Precision (RSD, %; n=3)
	ng/g		r^2	1 (mg/kg)	10 (mg/kg)	50 (mg/kg)	1 mg/kg	
salicylic acid hexoside	0.060	0.18	0.991	94.6	105.8	110.7	5	9
caffeic acid hexoside	0.050	0.15	0.945	94.9	100.5	100.6	2	10
dihydroferulic acid	0.030	0.09	0.923	100.4	112.0	99.7	8	8
kaempferol-3-dihexoside	0.040	0.12	0.994	95.8	100.8	102.8	4	7
sinapinic acid hexose	0.040	0.12	0.991	99.1	101.9	104.2	6	7
feruloyl quinic acid	0.030	0.09	0.99	102.8	110.5	99.4	4	7
caffeoyl quinic acid	0.030	0.09	0.991	111.9	100.8	99.7	5	9
caffeic acid	0.060	0.18	0.998	99.7	99.1	100.8	6	7
caffeoyl shikimate acid	0.040	0.12	0.999	100.7	99.7	98.9	5	9
hyperoside	0.030	0.09	0.991	111.9	110.8	106.5	8	8
rutin	0.050	0.15	0.999	100.3	99.7	100.9	6	7
coumaroyl diglucoside	0.050	0.15	0.995	99.1	100.7	106.4	6	8
ferulic acid	0.030	0.09	0.991	100.5	98.6	104.6	2	10
luteolin-7-O-glucoside	0.060	0.18	0.995	101.7	98.6	103.9	7	10
quercetin rhamnoside	0.050	0.15	0.995	99.7	101.6	102.9	3	7
dicaffeoyl quinic acid	0.040	0.12	0.999	101.6	100.7	99.6	8	7
coumaric acid	0.04	0.12	0.999	115.9	101.8	102.7	8	4
